# Tuning the Optical Properties of CsPbBr_3_ Nanocrystals by Anion Exchange Reactions with CsX Aqueous Solution

**DOI:** 10.1186/s11671-018-2592-4

**Published:** 2018-06-20

**Authors:** Anping Yan, Yunlan Guo, Chao Liu, Zhao Deng, Yi Guo, Xiujian Zhao

**Affiliations:** 1 State Key Laboratory of Silicate Materials for Architectures, Wuhan University of Technology, 122 Luoshi Road, Hongshan, Wuhan, 430070 China; 2 State Key Laboratory of Advanced Technology for Materials Synthesis and Processing, Wuhan University of Technology, 122 Luoshi Road, Hongshan, Wuhan, 430070 China; 3 Materials Research and Test Center, Wuhan University of Technology, 122 Luoshi Road, Hongshan, Wuhan, 430070 China

**Keywords:** CsPbBr_3_ nanocrystals, Anion exchange, CsX aqueous solution, Ultrasonication, Photoluminescence

## Abstract

Topotactic anion exchange has been developed to tune the composition and band gap energies of cesium lead halide (CsPbX_3_) perovskite nanocrystals (NCs). However, current anion exchange methods either require harsh conditions or take a long time to realize substantial substitution. Here, we present a method to modulate the composition of colloidal CsPbBr_3_ NCs through ultrasonication-assisted anion exchange with CsX (X = Cl, I) solution. Efficient anion exchange of CsPbBr_3_ NCs with Cl^−^ or I^−^ is realized with substitution ratio up to 93% and preservation of the pristine shape and structure of CsPbBr_3_ NCs. This anion exchange results in tunable emission, covering the whole visible spectral range, with relatively high photoluminescence quantum yield, narrow emission bandwidths, and good stability. This work provides a facile and efficient way to engineer the properties of halide perovskite NCs and has great potential for large-scale production of compositionally diverse perovskite NCs.

**Electronic supplementary material:**

The online version of this article (10.1186/s11671-018-2592-4) contains supplementary material, which is available to authorized users.

## Background

All-inorganic CsPbX_3_ (X=Cl, Br, and I) perovskite nanocrystals (NCs) have gained significant attention owing to their high photoluminescence (PL) quantum yield (QY) [1], narrow emission line width [2], defect tolerance [3, 4], and wide range of band gaps tunable by control of both composition [5, 6] and morphology [7–9]. The promise of CsPbX_3_ NCs has been examined in the context of their applications in optoelectronic devices, such as light-emitting diodes [10–14], Photodetectors [15, 16], lasing [17], and photovoltaics [18, 19].

Especially, the capability of anion exchange of CsPbX_3_ NCs has opened the door to many interesting applications of these materials. A broad range of tunable PL from CsPbX_3_ NCs could be obtained via a simple post-synthetic procedure, where mixing the solution of NCs with the reactive anion precursors results in the formation of the anion-exchanged NCs with tunable bandgap [5, 6, 20–22]. The labile nature of the halide anions in the perovskite system is generally responsible for their facile exchange in perovskite NCs and other processes involving ion transport, such as long-range anion diffusion under weak perturbation [23, 24] and the phase segregation in the methylammonium lead-mixed halide system [25]. Due to the simple and wide tunability of the photophysical properties of perovskite NCs, anion exchange has been extensively explored using various sources of anions for different applications [26, 27]. Most of the reported anion exchange methods generally require presynthesized halide precursors, and the reactivity of the halide containing precursors determines the extent and efficiency of the anion exchange. It has been reported that anion exchange of CsPbBr_3_ NCs with low-active precursors, such as PbX_2_, takes a long time (~ 1 day or longer) to proceed, and complete exchange of Br anion with X has been shown to be difficult [5]. Halide-containing precursors such as oleylamine halides (OLAM-X) and tetrabutylammonium halides (TBA-X) are highly reactive [5–7], which makes the anion exchange process very efficient, and complete anion exchange can be achieved. However, these highly reactive precursors are toxic and the anion exchange processes need to be carried out under inert and anhydrous conditions. Therefore, a new method for efficient and green anion exchange of CsPbX_3_ NCs is still worth pursuing.

**Fig. 1:**
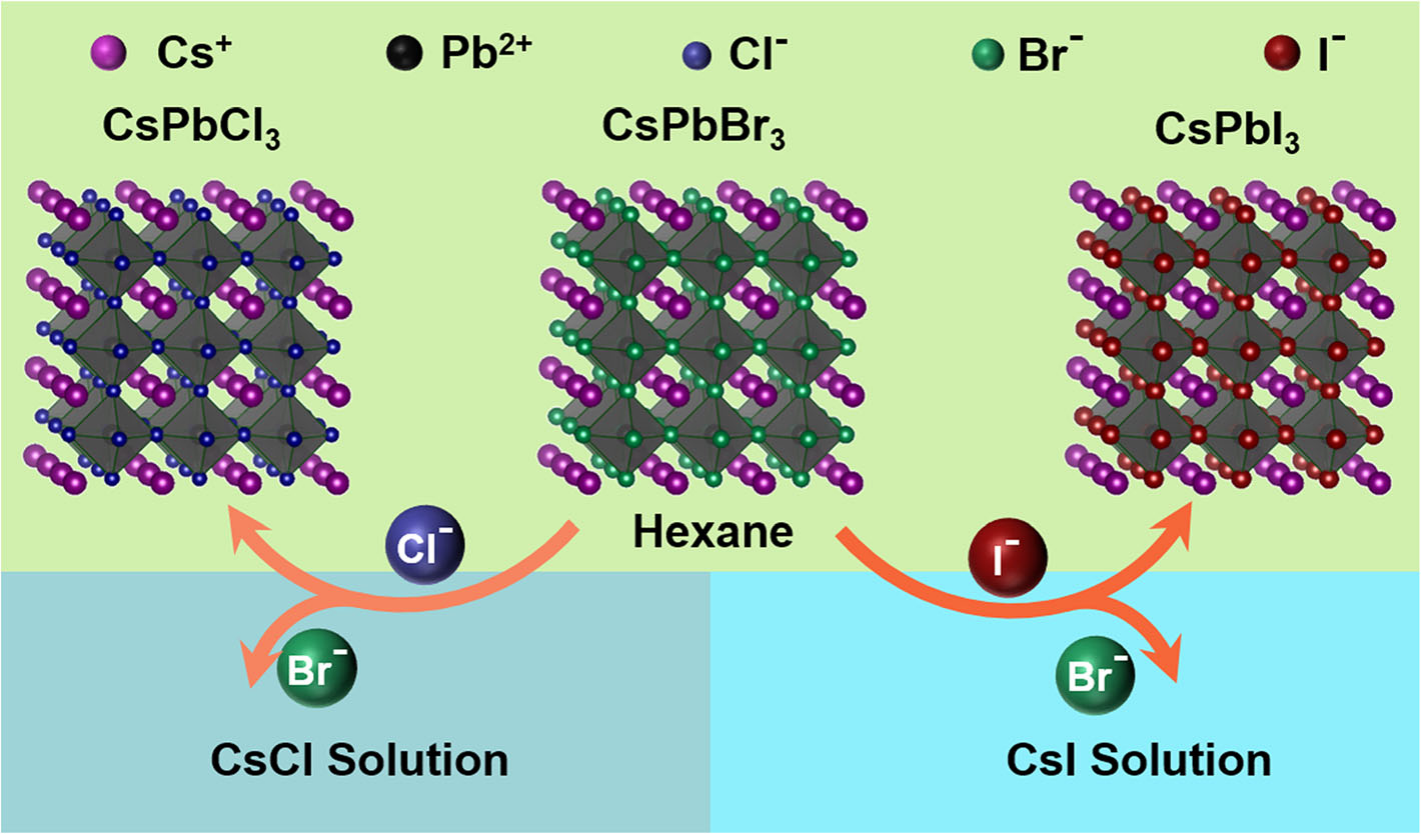
Illustration of the mechanism for anion exchange of perovskite nanocrystals in aqueous solutions

Recently, Yin et al. reported one efficient method to transform the nonluminescent Cs_4_PbX_6_ NCs into CsPbX_3_ NCs [28, 29]. These presynthesized Cs_4_PbX_6_ NCs were dispersed in nonpolar hexane, and the excess CsX was stripped and dissolved into the water through the interfacial reaction, and further etching of resultant CsPbBr_3_ NCs was prohibited by the interface between water and nonpolar solvent. Based on this report, we propose one facile anion exchange method to tune the composition and optical properties of the CsPbBr_3_ NCs. The presynthesized CsPbBr_3_ NCs are dispersed in hexane, and the anion exchange is realized through the interfacial reaction with CsX (X=Cl, I) aqueous solution assisted by ultrasonication. The extent and rate of the anion exchange reaction are controlled by ultrasonic time and CsX concentration. Compared to most of the reported anion exchange methods [5–7, 20–22], this anion exchange scheme is very facile and environment-friendly. The halide precursors for anion exchange can be dissolved into water instead of organic solution, and after anion exchange, halides dissolved in water can be easily separated by desiccation. Most importantly, the reaction can be carried out under normal condition, instead of the inert and anhydrous conditions. The proposed mechanism of the anion exchange of CsPbX_3_ NCs in CsX aqueous solutions is illustrated in Fig. 1 Br^−^ ions in the CsPbBr_3_ NCs exchanges with the Cl^−^ or I^−^ ions, resulting in the formation of CsPbX_3_ NCs. By adjusting the reaction time or the CsX aqueous solution, complete tunable composition of CsPbX_3_ NCs and emission covering the full visible spectral range with narrow band widths can be achieved.

## Methods

### Synthesis and Purification of CsPbBr_3_ NCs

CsPbBr_3_ NCs are synthesized following the method reported by Protesescu et al. [1]. In a typical experiment, 0.8 g of Cs_2_CO_3_(99.9%, Aldrich), 2.5 ml of oleic acid (OA; 90%, Aldrich) and 30 ml of octadecene (ODE; 90%, Aldrich) are added into a 100-ml 3-neck flask, degassed at room temperature for 30 min, and then dried for 1 h at 120 °C under Ar until all Cs_2_CO_3_ reacted with OA. 0.136 g of PbBr_2_ (99.9%, Aldrich), 2 mL of oleylamine (OALM; Aldrich, 80–90%), 1.5 mL of OA, and 8 mL ODE are added to a 25-mL 3-neck round bottom flask. The solution is evacuated and refilled with Ar followed by heating to 120 °C for 30 min. The solution is heated to 180 °C and kept for another 10 min. Then the Cs-oleate (1 mL) is injected and after 10 s the solution is cooled with an ice bath. The NCs are precipitated with acetone (AR, Sinopharm) and then centrifuged followed by dissolving in hexanes (AR, Sinopharm).

### Anion Exchange Reactions

5-ml of CsX (1 mol/L, 0.2 mol/L, X=Cl, I) aqueous solution is loaded in a 25-mL glass bottle and 3 ml of CsPbBr_3_ NCs/hexane solution (4.5 mmol/L in Br^‐^) is dropped inside, and then by bath-sonication (KQ-50B, ultrasonic cleaner) at a power of 50 W (total power) for some time. After ultrasonication, the system is then kept undisturbed for 5 min, and then the organic phase layer is collected (CsX aqueous solution can be reused by purification). Afterwards, the product is centrifuged at 2500 rpm for 5 min to discard the precipitates.

### Characterization

The phases of the products are examined by X-ray powder diffractometer (D8 Advance, Bruker) and high-resolution transmission electron microscope (HR-TEM, JEM 2100F, JEOL, Japan) operating at 200 kV. Absorption spectra of the colloidal CsPbX_3_ NCs are recorded using an U*V*/Vis/NIR spectrophotometer (UV3600, Shimadzu, Japan).

### Photoluminescence Measurements

Photoluminescence (PL) spectra of the colloidal CsPbX_3_ NCs are collected by a Photo Technology International (PTI) QM/TM/NIR spectrophotometer with a 914 photomultiplier detection system and using a 75-W xenon lamp as the excitation source. All the optical measurements are carried out at room temperature. 400 nm of light is used as the excitation source for all the CsPbX_3_ NCs, except 360 nm for CsPb(Br/Cl)_3_ NCs. Following the method proposed by Prato et al. [5], four measurements are performed for PLQY: (i) the sample emission (SEM) that collects the photons emitted by the sample, (ii) the blank emission(BEM), which is a measurement performed with the cuvette containing only the solvent (blank) in the same spectral range used for the SEM measurement, (iii) the sample excitation (SEX), which records the photons at the pumping wavelength that are not absorbed by the sample, and (iv) the blank excitation (BEX), which records the photons at the pumping wavelength going through the blank. The photoluminescence quantum yield PLQY is then calculated as

$$ \mathrm{PLQY}\left(\%\right)=\frac{\mathrm{SEM}\hbox{-} \mathrm{BEM}}{\mathrm{BEX}\hbox{-} \mathrm{SEX}}\times 100 $$

Any reabsorption correction factor is neglected in our calculation of the PLQY, since the solutions investigated are diluted to the point that reabsorption of the PL could be neglected.

### Stability Test

The perovskite NCs are dispersed in hexane and sealed into glass bottles, which are kept under ambient condition for several weeks. Changes in the absorption and PL of these anion-exchanged perovskite NCs are recorded at an interval of 7 days.

## Results and Discussion

**Fig. 2:**
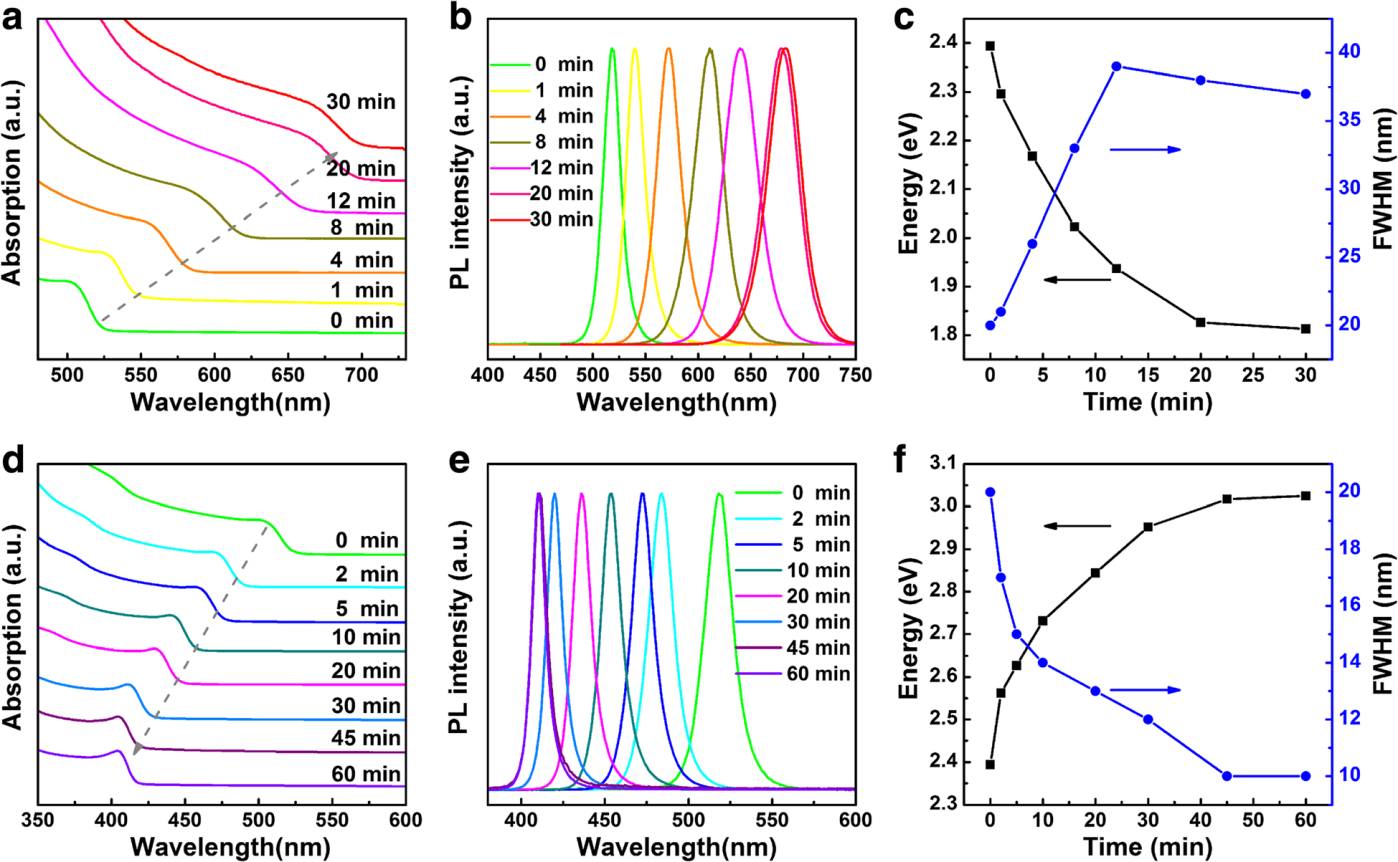
Ultrasonication time dependent absorption and emission of CsPbBr_3_ NCs exchanged with CsX aqueous solutions (1 mol/L). **a** Absorption spectra, **b** emission spectra, and **c** emission peak energy (black square) and emission bandwidth (blue circle) of CsPbBr_3_ NCs exchanged in CsI aqueous solution. **d** Absorption spectra, **e** emission spectra, and **f** emission peak energy (black square) and emission bandwidth (blue circle) of CsPbBr_3_ NCs exchanged in CsCl aqueous solution

Figure 2 summarizes the continuous changes in the absorption and emission spectra of mixed anion CsPbX_3_ NCs obtained through ion-exchange with CsI aqueous solution (Fig. 2a, b) and CsCl aqueous solution (Fig. 2d,e). The time-trace and full width at half maximum (FWHM) of the emission peaks are also shown for both reactions (Fig. 2c,f). Absorption and emission peaks of CsPbX_3_ NCs showed obvious red shift with anion exchange with CsI solution, indicating the exchange of bromide with iodide and formation of CsPb(Br/I)_3_ NCs. Upon 30-min ultrasonication, absorption and emission peaks stabilize at 675 nm (Fig. 2a) and 685 nm (Fig. 2b), respectively. With CsCl solution, both absorption and emission peaks of CsPb(Br/Cl)_3_ NCs show gradual blue shift upon continuous ultrasonication. With ultrasonication, the absorption peak shifts to 405 nm (Fig. 2d) and the emission peak shifts to 411 nm (Fig. 2e) within 45 min and stabilizes. This process allows the formation of CsPbX_3_NCs with tunable band gap energies (Fig. 2c, f) and emission spanning over the whole visible spectral range. It has to be pointed out that the FWHM of CsPb(Br/I)_3_ NCs gradually increases from 20 nm to a maximal value of 39 nm (Fig. 2c), while the FWHM of CsPb(Br/Cl)_3_ NCs monotonically decreased from 20 to 10 nm (Fig. 2f). These changes in the FWHM values show that the size dispersion of the pristine CsPbBr_3_ NCs is largely maintained.

**Fig. 3:**
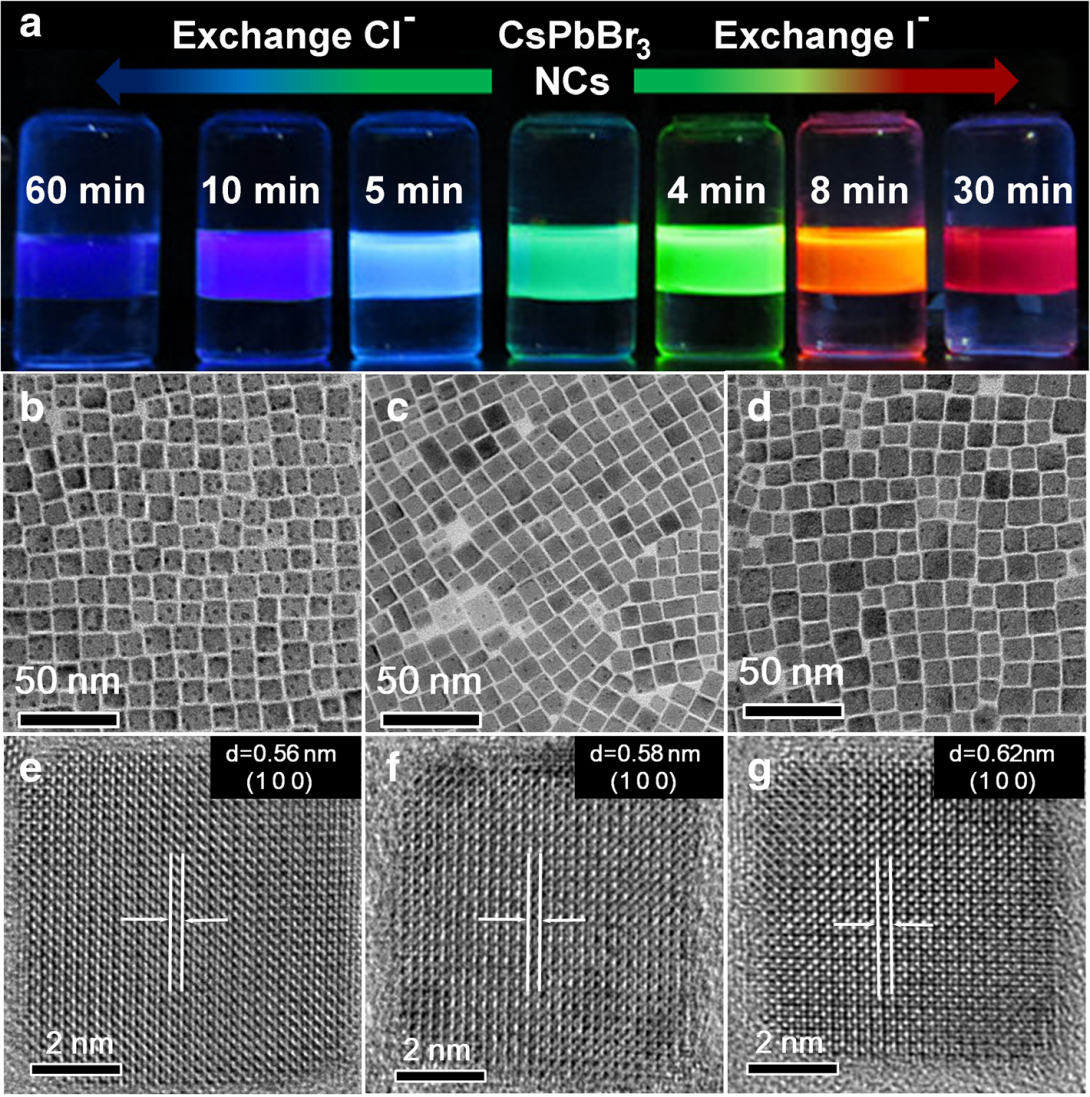
**a** Emission photograph of perovskite NCs exchanged with 1 mol/L CsX aqueous solution under 365-nm light illumination. TEM and HR-TEM images of CsPb(Br/Cl)_3_ NCs obtained from 60 min exchange (**b**, **e**), pristine CsPbBr_3_ NCs (**c**, **f**), and CsPb(Br/I)_3_ NCs obtained from 30 min exchange (**d**, **g**)

Emission photograph of the anion-exchanged perovskite NCs is shown in Fig. 3a. Upon exchange with CsCl or CsI aqueous solution, emission color of the NCs gradually changes to blue or red. Most importantly, size and morphology of the CsPbBr_3_ NCs template are preserved during the anion-exchange process (Fig. 3b–d). As confirmed by the TEM images, after the anion exchange, average sizes of CsPb(Br/Cl)_3_ NCs with sonication time of 60 min (Fig. 3b) and CsPb(Br/I)_3_ NCs with sonication time of 30 min (Fig. 3d) are almost the same as the pristine CsPbBr_3_ NCs (Fig. 3c), and the shape still remains as cubic. CsPbX_3_ NCs obtained with other sonication time (Additional file 1: Figure S1 for CsI aqueous solution and Additional file 1: Figure S3 for CsCl aqueous solution) show the same cubic shape, although after exchange of Br^−^ ions with I^−^ ions their size increased slightly from (9.6 ± 1.3) to (11.1 ± 1.5) nm (Additional file 1: Figure S2), whereas the exchange with Cl^−^ ions led to a slight decrease in size, to (8.2 ± 1.4) nm (Additional file 1: Figure S4). The selected area electron diffraction pattern (Additional file 1: Figure S5) confirms that these CsPbBr_3_ NCs and exchanged CsPbX_3_ NCs all had the same cubic structure with space group of $Pm\overline{3}m$(221). The HR-TEM images (Fig. 3e–f) evidenced the high quality of the pristine CsPbBr_3_ NCs (Fig. 3f), the exchanged CsPb(Br/Cl)_3_ NCs (Fig. 3e) and CsPb(Br/I)_3_NCs (Fig. 3g). These results shows that the anion exchange with CsX aqueous solution does not deteriorate or etch the perovskite NCs. Successful exchange of Br^−^ ions with Cl^−^ or I^−^ ions is further confirmed by the changes in the lattice distance shown in Fig. 3e–g. For the perovskite NCs obtained through exchange with CsCl aqueous solution for 60 min, the (100) lattice constant is found to be 0.56 nm, almost identical to that of CsPbCl_3_ NCs (JCPDF No.: 75-0411). For those NCs exchanged with CsI aqueous solution for 30 min, the (100) lattice constant increases from 0.583 nm of CsPbBr_3_ NCs (JCPDF No.: 54-0752) to 0.615 nm, close to that of CsPbI_3_ NCs [5–7]. For CsPb(Br/I)_3_ NCs, the (100) lattice plane distance increases from 0.583 nm of CsPbBr_3_ NCs to 0.591, 0.6, and 0.615 nm as the sonication time increases from 0 to 4, 8, and 30 min, respectively (Additional file 1: Figure S1). For CsPb(Br/Cl)_3_ NCs, the (100) lattice plane distance decreased from 0.583 nm of CsPbBr_3_ NCs to 0.575, 0.57, and 0.561 nm, as the sonication time increases from 0 to 5, 10, and 60 min, respectively (Additional file 1: Figure S3). X-ray diffraction pattern also shows that all the diffraction peaks of the CsPbX_3_ NCs can be assigned to cubic perovskite, and they gradually shift towards higher angles approaching that of CsPbCl_3_ NCs, and lower angles approaching that of CsPbI_3_ NCs when exchanged with CsCl and CsI aqueous solution, respectively (Additional file 1: Figure S6).

Actual compositions of the exchanged perovskite NCs analyzed using energy dispersive X-ray spectroscopy (EDX) are listed in Table 1, along with the measured PLQY and emission peak energy. With continuous anion exchange with CsX aqueous solution, the Br^−^ to Cl^−^ substitution ratio can reach 93% in CsPb(Br/Cl)_3_ NCs, and the Br^−^ to I^−^ substitution ratio can reach 90%. These substitution ratios are comparable to those achieved with highly reactive precursors such as OLAM-X and TBA-X [5–8], demonstrating that anion exchange through CsX aqueous solution was also a powerful route to tune the chemical composition of CsPbX_3_ NCs. With anion exchange, the PLQY of CsPb(Br/Cl)_3_ NCs firstly increases from 76% of the pristine CsPbBr_3_ NCs to 85% of the CsPbBr_2.3_Cl_0.7_ NCs, and then rapidly drops to 32% (CsPbBr_2.0_Cl_1.0_ NCs) and 5% (CsPbBr_0.2_Cl_2.8_ NCs). The PLQY of the CsPb(Br/I)_3_ NCs monotonically declines from 76% of the pristine CsPbBr_3_ NCs to 31% of CsPbBr_0.3_I_2.7_ NCs. This trend is in line with that reported by Pellet et al. [30]. It has to be pointed out that PLQY of the CsPbX_3_ NCs obtained through the longest sonication is comparable to those of directly synthesized CsPbI_3_ and CsPbCl_3_ NCs [30–32], further confirming that anion exchanged through CsX aqueous solution does not lead to the deterioration of the optical properties of the CsPbX_3_ NCs. Even though these CsPbX_3_ NCs are exchanged with aqueous solution, they still maintain relative good room temperature stability when stored in hexane, mainly due to the low solubility of water in hexane (9.5 mg/L) [28]. For CsPb(Br/Cl)_3_ NCs obtained through 45 min sonication, the PL intensity decreased to ~ 30% of the original intensities within 4 weeks (Additional file 1: Figure S7a). While for CsPb(Br/I)_3_ NCs obtained through 20 min sonication, only 5% of the original PL intensities was retained within 4 weeks (Additional file 1: Figure S7b).

**Table 1:** Composition, emission energy, and PLQY of CsPbBr_3_NCs exchanged under different conditions

Aqueous solution	Sonication time (min)	EDX composition	PL energy (eV)	PL QYs (%)
1 mol/L CsCl	60	CsPbBr_0.2_Cl_2. 8_	3.04	5
	10	CsPbBr_2.0_Cl_1.0_	2.74	32
	5	CsPbBr_2.3_Cl_0.7_	2.62	85
Pristine	0	CsPbBr_3_	2.39	76
1 mol/L CsI	4	CsPbBr_2.5_I_0.5_	2.25	52
	8	CsPbBr_2.2_I_0.8_	2.03	46
	30	CsPbBr_0.3_I_2.7_	1.83	31

**Fig. 4:**
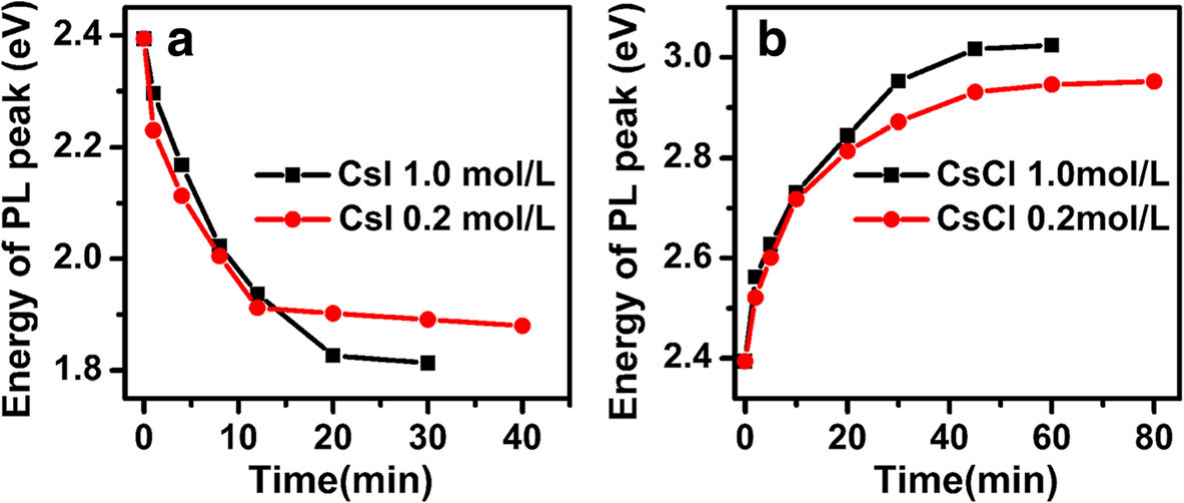
Emission peak energies of CsPbX_3_ NCs exchanged with 1.0 and 0.2 mol/L **a** CsI and **b** CsCl aqueous solution

This facile ultrasonication-assisted anion exchange with CsX aqueous solution is mainly driven by the large concentration of CsX in the aqueous solution. Anion-exchange of CsPbBr_3_ NCs with octadecylamine halides(ODA-X) and lead halides (PbX_2_) salts was found to be slow and incomplete [5], due to the low solubility of these compounds in the nonpolar toluene solvent. The relatively large solubility of OLAM-X and TBA-X in toluene makes the anion exchange of CsPbBr_3_ NCs very fast and complete [5–8]. The fast anion exchange and wide tunable spectral range of absorption and emission reported in this work are mainly ascribed to the large solubility of CsX in water (1865 g/L for CsCl and 440 g/L for CsI in water) [28], which provide the large driving force for the anion exchange. To further confirm the effect of CsX aqueous solution concentration on the anion exchange of CsPbX_3_ NCs, CsPbBr_3_ NCs are exchanged with CsX aqueous solution of 0.2 mol/L (Fig. 4). It is found that the PL peak energy of CsPb(Br/I)_3_ NCs gradually stabilized at 1.88 eV (Fig. 4a) with 40-min sonication, and the PL peak energy of CsPb(Br/Cl)_3_ NCs gradually approaches at 2.95 eV (Fig. 4b) even with 80-min ultrasonication. The ultrasonication time dependence of the emission peak energy shows that concentration of the CsX aqueous solution does not affect the speed at the early stage, but determines the final composition of the CsPbX_3_ NCs, providing a new route to precisely control the composition of the exchanged CsPbX_3_ NCs.

## Conclusions

In conclusion, we report here a simple and environment-friendly ultrasonication assisted anion-exchange of CsPbBr_3_ NCs with CsX aqueous solution. This anion-exchange happens at the interface of the CsX aqueous solution and hexane. By carefully selecting the anion-exchange condition, more than 90% of the Br^−^ ions in CsPbBr_3_ NCs can be substituted by X^−^ ions, while maintaining the shape and structure of the pristine CsPbBr_3_ NCs. Both absorption and emission of these exchanged CsPbX_3_ NCs can span the full visible spectral range, with a relatively high PLQY and stability. This anion-exchange method provides another facile route to modulate the chemical compositions and optical properties of CsPbX_3_ NCs.

## Additional file

Additional file 1: Additional XRD patterns, TEM images, and PL spectra (DOCX 4026 kb)

## Abbreviations

EDX: Energy-dispersive X-ray spectroscopy
NCs: Nanocrystals
OA: Oleic acid
OALM: Oleylamine
ODE: Octadecene
PL: Photoluminescence
TBA: Tetrabutylammonium
TEM: Transmission electron microscope
XRD: X-ray diffraction
