# Supplementary material for: Tuning the Optical Properties of CsPbBr3 Nanocrystals by Anion Exchange Reactions with CsX Aqueous Solution
Source: Nanoscale Res Lett. 2018 Jun 20;13:185. doi: 10.1186/s11671-018-2592-4 (PMC6010365; doi:10.1186/s11671-018-2592-4)
Supplement: Supplementary file 1 — Additional XRD patterns, TEM images, and PL spectra (DOCX 4026 kb) [file 11671_2018_2592_MOESM1_ESM.docx]

Additional file 1 for

**Tuning the Optical Properties of CsPbBr_3_ Nanocrystals by Anion Exchange Reactions with CsX Aqueous Solution**

Anping Yan^a^, Yunlan Guo^a^, Chao Liu, *^a^ Zhao Deng,*^b^ Yi Guo^c^ and Xiujian Zhao*^a^

^a^State Key Laboratory of Silicate Materials for Architectures, Wuhan University of Technology, 122 Luoshi Road, Hongshan, Wuhan 430070, China

^b^State Key Laboratory of Advanced Technology for Materials Synthesis and Processing, Wuhan University of Technology, 122 Luoshi Road, Hongshan, Wuhan 430070, China

^c^Materials Research and Test Center, Wuhan University of Technology, 122 Luoshi Road, Hongshan, Wuhan 430070, China

**Supporting Figures**

**
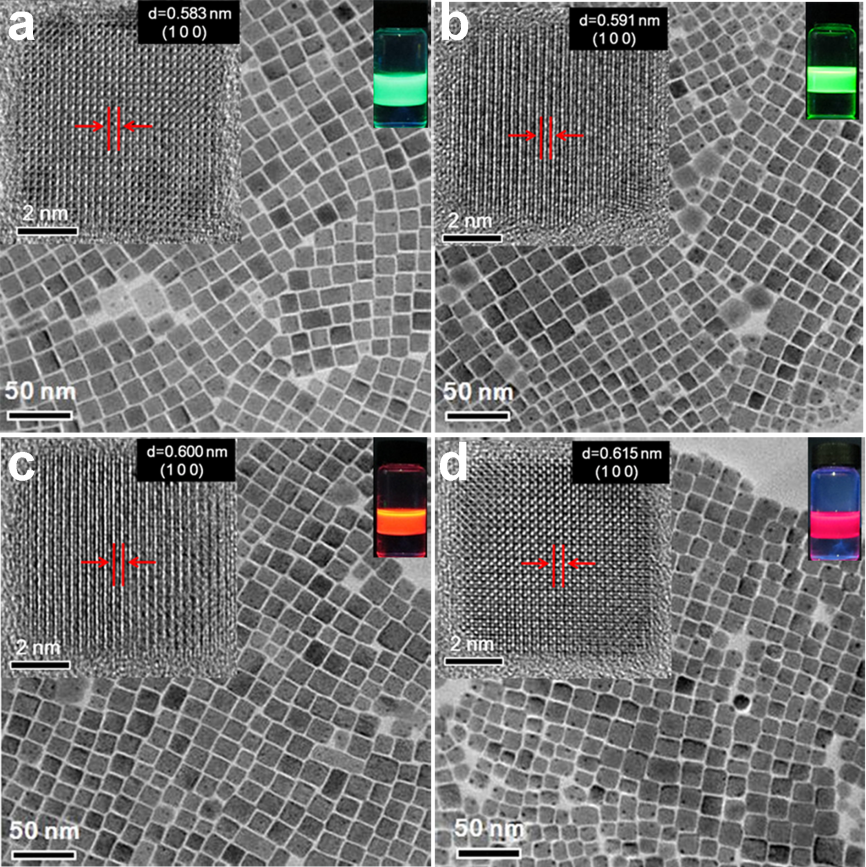
**

Fig. S1 TEM images of CsPb(Br/I)_3_ NCs obtained through anion exchange with CsI aqueous solution for various ultrasonication time. (a) 0 min, (b) 4 min, (c) 8 min, (d) 30 min. Insets are the HR-TEM image of the corresponding NCs and their emission photograph under 365 nm UV light excitation.


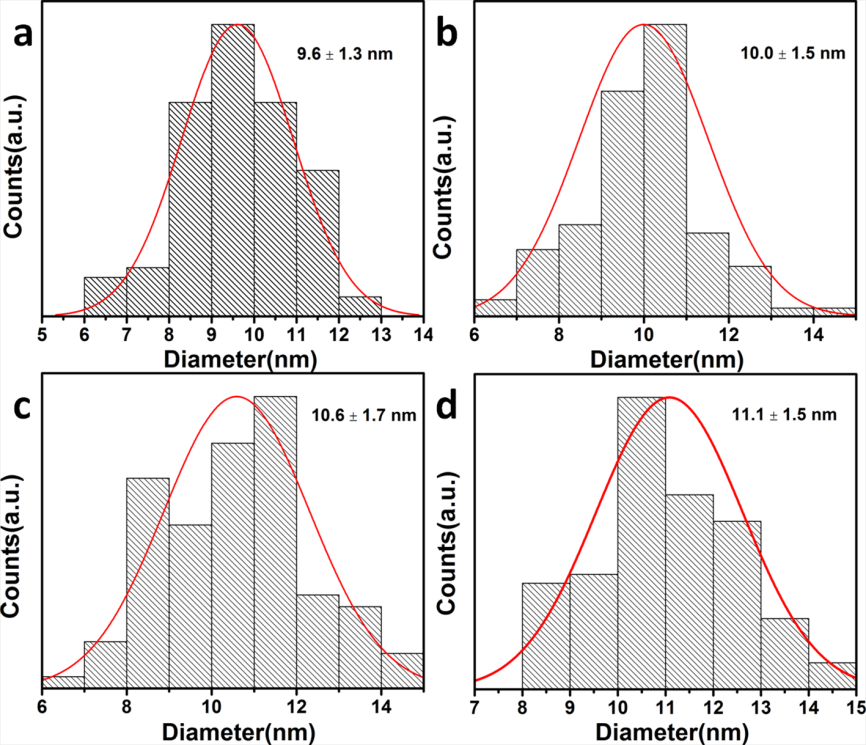


Fig. S2 Size distribution of CsPb(Br/I)_3_ NCs obtained through anion exchange with CsI aqueous solution for various ultrasonication time. (a) 0 min, (b) 4 min, (c) 8 min, (d) 30 min, as determined by size analysis on more than 100 NCs.


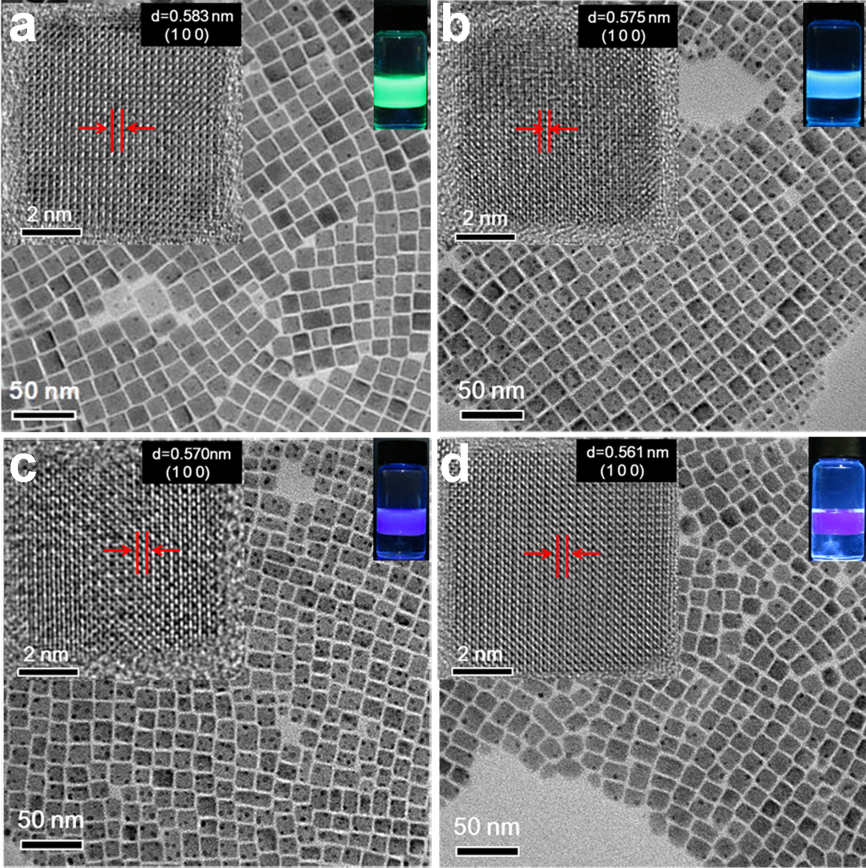


Fig. S3 TEM images of CsPb(Br/Cl)_3_ NCs obtained through anion exchange with CsCl aqueous solution for various ultrasonication time. (a) 0 min, (b) 5 min, (c) 10 min, (d) 60 min. Insets are the HR-TEM image of the corresponding NCs and their emission photograph under 365 nm UV light excitation.


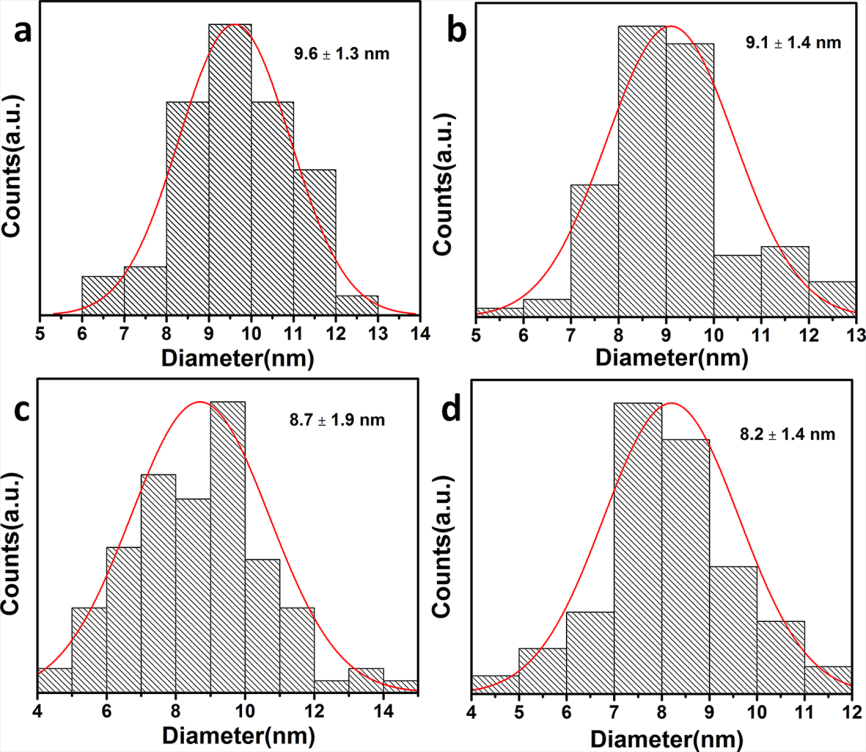


Fig. S4 Size distribution of CsPb(Br/Cl)_3_ NCs obtained through anion exchange with CsCl aqueous solution for various ultrasonication time. (a) 0 min, (b) 5 min, (c) 10 min, (d) 60 min, as determined by size analysis on more than 100 NCs.


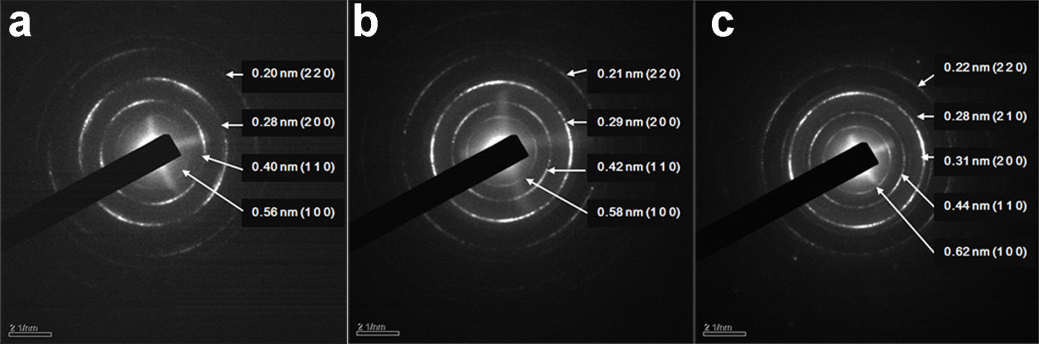


Fig. S5 The selected area electron diffraction patterns of CsPbX_3_NCs. (a) CsPb(Br/Cl)_3_ NCs with 60 min ultrasonication, (b) pristine CsPbBr_3_ NCs, (c) CsPb(Br/I)_3_ NCs with 30 min ultrasonication.





Fig. S6 XRD patterns of pristine CsPbBr_3_ NCs (green curve), colloidal CsPb(Br/Cl)_3_ NCs with 30 min (blue curve) and 10 min (olive curve) ultrasonication with 1 mol/L CsCl solution, and colloidal CsPb(Br/I)_3_ NCs with 5 min (yellow curve) and 20 min (red curve) ultrasonication with 1 mol/L CsI solution.


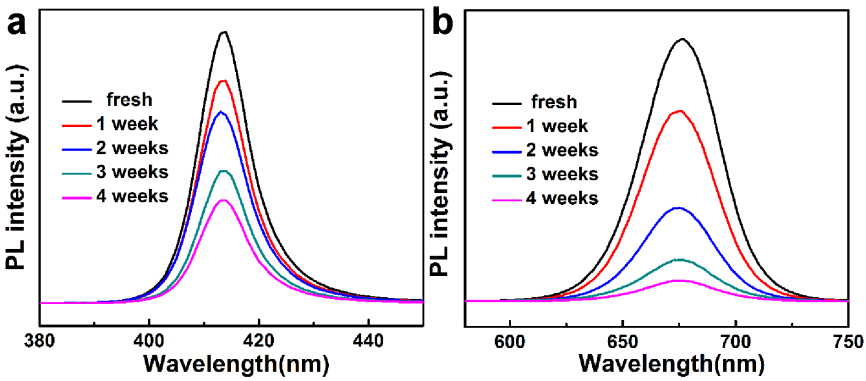


Fig. S7 Stability of emission intensities of (a) CsPb(Br/Cl)_3_ NCs (45 min ultrasonication) and

(b) CsPb(Br/I)_3_ NCs (20 min ultrasonication).
